# Platelets Induce Apoptosis during Sepsis in a Contact-Dependent Manner That Is Inhibited by GPIIb/IIIa Blockade

**DOI:** 10.1371/journal.pone.0041549

**Published:** 2012-07-26

**Authors:** Matthew Sharron, Claire E. Hoptay, Andrew A. Wiles, Lindsay M. Garvin, Mayya Geha, Angela S. Benton, Kanneboyina Nagaraju, Robert J. Freishtat

**Affiliations:** 1 Division of Critical Care Medicine, Children’s National Medical Center, Washington, D.C., United States of America; 2 Department of Pediatrics, George Washington University School of Medicine and Health Sciences, Washington, D.C., United States of America; 3 Department of Integrative Systems Biology, George Washington University School of Medicine and Health Sciences, Washington, D.C., United States of America; 4 Research Center for Genetic Medicine, Children’s National Medical Center, Washington, D.C., United States of America; 5 Department of Microbiology, Immunology, and Tropical Medicine, George Washington University School of Medicine and Health Sciences, Washington, D.C., United States of America; 6 Division of Emergency Medicine, Children’s National Medical Center, Washington, D.C., United States of America; Fundação Oswaldo Cruz, Brazil

## Abstract

**Purpose:**

End-organ apoptosis is well-described in progressive sepsis and Multiple Organ Dysfunction Syndrome (MODS), especially where platelets accumulate (e.g. spleen and lung). We previously reported an acute sepsis-induced cytotoxic platelet phenotype expressing serine protease granzyme B. We now aim to define the site(s) of and mechanism(s) by which platelet granzyme B induces end-organ apoptosis in sepsis.

**Methods:**

End-organ apoptosis in murine sepsis (i.e. polymicrobial peritonitis) was analyzed by immunohistochemistry. Platelet cytotoxicity was measured by flow cytometry following 90 minute *ex vivo* co-incubation with healthy murine splenocytes. Sepsis progression was measured via validated preclinical murine sepsis score.

**Measurements and Main Results:**

There was evident apoptosis in spleen, lung, and kidney sections from septic wild type mice. In contrast, there was a lack of TUNEL staining in spleens and lungs from septic granzyme B null mice and these mice survived longer following induction of sepsis than wild type mice. In co-incubation experiments, physical separation of septic platelets from splenocytes by a semi-permeable membrane reduced splenocyte apoptosis to a rate indistinguishable from negative controls. Chemical separation by the platelet GPIIb/IIIa receptor inhibitor eptifibatide decreased apoptosis by 66.6±10.6% (p = 0.008). Mice treated with eptifibatide *in vivo* survived longer following induction of sepsis than vehicle control mice.

**Conclusions:**

In sepsis, platelet granzyme B-mediated apoptosis occurs in spleen and lung, and absence of granzyme B slows sepsis progression. This process proceeds in a contact-dependent manner that is inhibited *ex vivo* and *in vivo* by the platelet GPIIb/IIIa receptor inhibitor eptifibatide. The GPIIb/IIIa inhibitors and other classes of anti-platelet drugs may be protective in sepsis.

## Introduction

Despite several decades worth of advances in antimicrobials, critical care, and organ support modalities, mortality rates from septic shock/severe sepsis have remained at about 30–40% [1]. In fact, sepsis is responsible for 215,000 U.S. deaths annually, which is akin to mortality from acute myocardial infarction [1], making it the 10th leading cause of death [2]. The frequent precursor to mortality from sepsis is Multiple Organ Dysfunction Syndrome (MODS), with increased numbers of failing organs associated with higher mortality 3–5. Many of these failing organs – in particular lung, intestine, vascular endothelium, and lymphoid tissue – show marked apoptotic cell death during sepsis [6]–[9]. We recently identified a potential etiologic factor for sepsis-related end-organ apoptosis: Acute sepsis-induced alterations in the megakaryocyte-platelet transcriptional axis result in strongly cytotoxic platelets expressing the potent serine protease granzyme B in mice and humans [10].

It is notable that platelets accumulate in the microvasculature of many of these commonly failing apoptotic end organs in sepsis (e.g. lung, liver, intestine, and spleen) [11]–[14], and platelet derived microparticles are cytotoxic to a variety of cell types including vascular endothelium [15]–[17] and smooth muscle [17]. Therefore, we hypothesized that septic platelet-induced apoptosis occurs in both non-lymphoid and lymphoid organs and that this cytotoxicity is independent of direct platelet-target cell contact (i.e. microparticle-mediated).

## Methods

### Ethics Statement

This study was carried out in strict accordance with the recommendations in the Guide for the Care and Use of Laboratory Animals of the National Institutes of Health. The Children’s National Medical Center Institutional Animal Care and Use Committee approved all experiments (IACUC approval # 207-07-08 and # 280-11-08). All surgery was performed under isoflurane and nitric oxide anesthesia, and all efforts were made to minimize suffering.

### Animals

Wild type (i.e. C57BL6), perforin null (i.e. C57BL/6-*Pfp^tm1Sdz^*), and granzyme B null mice (i.e. B6.129S2-*GzmB^tm1Ley^*) (Jackson Laboratories, Bar Harbor, ME) were housed and bred in a conventional animal facility.

### Experimental Sepsis and Sample Collection

For most experiments, polymicrobial peritonitis and experimental sepsis was induced via a moderate-severity cecal ligation and puncture (CLP) in 7 to 10-week-old male mice as we and others have previously described [10], [18], [19]. For natural history mortality studies, we used a severe CLP model for rapid time to death to minimize animal discomfort [19]. For the *in vivo* trial of eptifibatide, polymicrobial sepsis was induced in mice using the cecal slurry (CS) method, as described by Wynn et al [20]. Briefly, for CS preparation a mouse was euthanized and a midline incision was made to isolate the cecum. The cecal contents were homogenized and suspended in 5% dextrose at a final concentration of 80 mg/mL. The resulting slurry was frozen at -80°C and thawed within one week for intraperitoneal (IP) administration to recipient mice (7 to 10-week-old males) at a dose of 2 mg of cecal content per gram of mouse weight. Sham mice received an IP injection of 5% dextrose.

Mice were scored post-CLP or post-CS injection at 2-hour intervals, starting at either 12 or 16 hours, using a 15-point validated murine sepsis severity measure [21], [22]. Mice were sacrificed when a score of 10 (associated with >90% imminent mortality) was reached or at 72 hours. For non-mortality experiments, mice were sacrificed 18 hours post-surgery. At the time of sacrifice, intra-cardiac blood was drawn into sodium citrate (Becton-Dickinson, Franklin Lakes, NJ) and centrifuged for platelet-rich plasma at 770 rpm for 20 minutes at 25°C. Platelets were isolated by centrifugation and filtered through a 10 mL sepharose 2B gel column [23]. Platelet concentrations were measured and standardized using a manual hemocytometer. We confirmed platelet isolates to be pure and not platelet-leukocyte aggregates based upon size by flow cytometry and lack of staining for CD45 (BD Pharmingen, San Diego, CA). Endotoxin levels were measured in plasma using ToxinSensor™ Chromogenic LAL Endotoxin Assay Kit (GenScript, Piscataway, NJ).

### Administration of Eptifibatide

Mice received intravenous injections of eptifibatide (15 µg/g of mouse weight) or PBS vehicle via tail vein injection at 12, 16, and 20 hours post-injection of cecal slurry. This dosing regimen was chosen based on the kinetics of platelet granzyme B expression we previously published [10] (i.e. expression peak after 12 hours) and the pharmacology of eptifibatide (i.e. plasma elimination half-life of approximately 2.5 hours). In addition, we planned for a maximum of three tail vein injections considering the vascular compromise of the septic animals and resulting difficulty with injections.

### Ex Vivo Platelet-Splenocyte Co-Incubation

Non-septic wild type spleens were firmly pressed between two glass slides to express splenocytes, which were isolated by centrifugation through Ficoll-Paque™ Plus (GE Healthcare Bio-Sciences Corporation, Piscataway, NJ). Splenocyte concentrations were measured and standardized using a manual hemocytometer and co-incubated *ex vivo* with platelets (from septic or healthy control mice) for 90 minutes at 37°C and 5% CO_2_ in complete Dulbecco’s Modified Eagle Medium (DMEM) (Invitrogen/GIBCO, Carlsbad, CA). For some experiments, a 0.4 µm semi-permeable membrane (Corning Inc., Corning, NY) was used to physically separate platelets from splenocytes. In other experiments, platelet-splenocyte contact was pharmacologically inhibited using anti-aggregatory pretreatment with GPIIb/IIIa inhibitor, eptifibatide (4 µg/mL; Bachem, Torrance, CA), or anti-CD62p antibody (3 µg/mL; clone RB40.34; BD Biosciences, San Jose, CA) for 15 minutes.

### Detection of Apoptosis

Splenocyte apoptosis in each experimental condition was quantified in cell suspensions by flow cytometry on a FACSCalibur™ (Becton, Dickinson and Company, San Jose, CA) and in tissue sections by immunohistochemistry on a Nikon Eclipse E 800 Microscope (Nikon Instruments Inc., Melville, NY) with a Spot RT Slider Camera (Diagnostic Instruments Inc., Sterling Heights, MI). Splenocyte suspension apoptosis was identified using FlowTACS™ (Trevigen, Gaithersburg, MD), a TUNEL-based assay for detection of DNA fragmentation. Positive controls were generated with staurosporine (Sigma Life Sciences, St Louis, MO). CD4+ fractions were identified by fluorophore-labeled antibody staining (clone L3T4; eBiosciences, San Diego, CA). We used a Sulforhodamine FLICA-Apoptosis Detection Kit Pan-Caspase Assay (Immunochemistry Technologies, Bloomington, MN) to measure activated caspases in apoptotic cells. Immunohistochemistry was performed on frozen heart, lung, kidney, spleen, and liver sections (4–7 µm) stained with the TUNEL-based TACS® 2 TdT In Situ Apoptosis Detection Kit (Trevigen, Gaithersburg, MD) according to the manufacturer’s instructions. Lung, spleen, and kidney sections were additionally stained with anti-CD41 (Rat anti-mouse CD41 antibody, Clone MWReg30, BD Pharmingen, San Diego, CA) to identify platelet accumulation. Apoptotic index was calculated for each tissue sample as described in Dmowski et al [24].

### Statistical Analyses

Flow cytometry data were analyzed using FlowJo 7.5 (Tree Star, Inc., Ashland, OR). Data were maintained in Microsoft Excel 2010 (Microsoft, Redmond, WA). Statistical significance was tested using paired and unpaired t-tests, Kaplan-Meier survival analyses, and Cox Proportional Hazard Regression in SPSS 20 (SPSS, Chicago, IL).

## Results

### Sepsis-Related Mortality is Reduced in the Absence of Granzyme B

**Figure 1:**
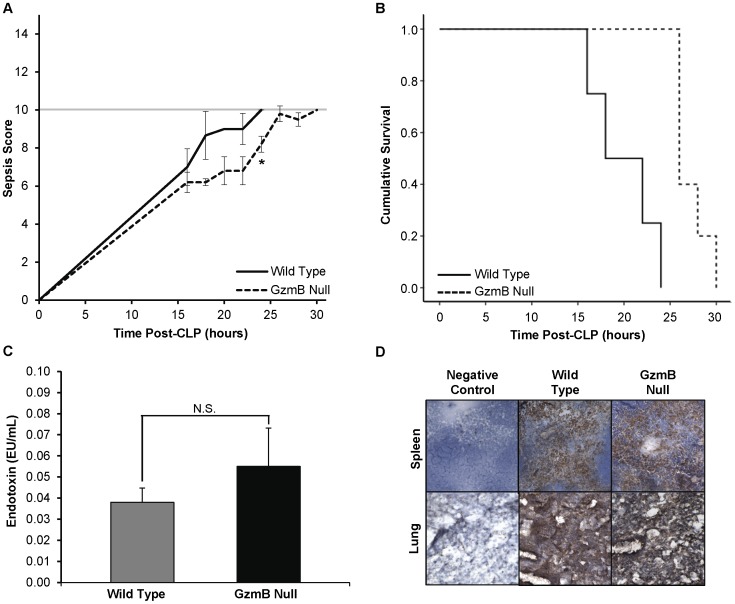
Sepsis survival and severity in wild type and granzyme B null mice in a rapidly fatal (severe) CLP model. **A.** Granzyme B null (−/−) mice had lower sepsis scores than wild type mice at every time point. For example, at 22 hours, the mean±SEM wild type score was 9.0±0.8 while the granzyme B null score was 6.8±0.7 (*p = 0.04) **B.** Kaplan-Meier survival curve for wild type and granzyme B null (−/−) mice in hours after CLP. Granzyme B null (−/−) mice survived longer following severe CLP than wild type mice (p = 0.0019 by Cox Proportional Hazard Regression). **C.** Endotoxin concentrations (EU/mL) were measured in granzyme B null and wild type mouse plasma. Differences between the two mouse strains were not statistically significant. **D.** Representative photomicrographs of lung and spleen in sepsis are shown. Platelet infiltration, assayed by CD41 (brown) staining, was visibly widespread and similar between wild type and granzyme B null mice in both organs. Photomicrographs were taken at 10X magnification.

Following CLP-induced polymicrobial sepsis (severe model), granzyme B null mice (n = 5) had lower sepsis scores than wild type mice (n = 4) at every time point. (Figure 1A) For example, at 22 hours, the mean ± SEM wild type score was 9.0±0.8 while the granzyme B null score was 6.8±0.7 (p = 0.04). At 24 hours post-CLP, the mortality rate of the granzyme B null mice was 0% while the mortality rate of the wild type mice was 100%. Kaplan-Meier survival analysis showed that granzyme B null mice survived longer following severe CLP than wild type mice (p = 0.0019 by Cox Proportional Hazard Regression). (Figure 1B) Granzyme B null and wild type mice did not differ according to plasma endotoxin concentrations (EU/mL). (Figure 1C).

### Sepsis-Induced Spleen and Lung Apoptosis Is Granzyme B-Dependent

**Figure 2:**
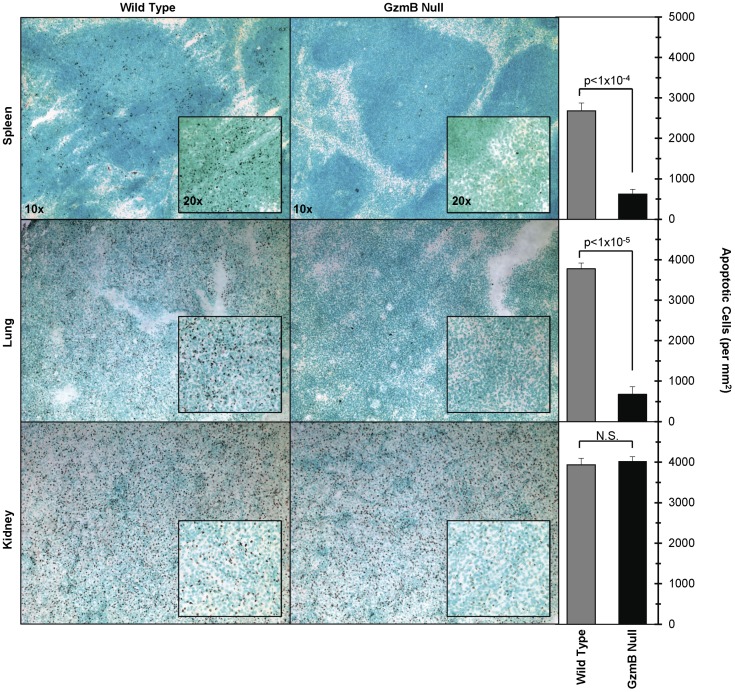
Platelet granzyme B apoptosis surveyed by TUNEL in spleen, lung, and kidney. Representative frozen sections of end organs (i.e. spleen (top), lung (middle), and kidney (bottom)) from wild type (left) and granzyme B null mice (right) were stained for apoptosis with a TUNEL-based assay (TACS® 2 TdT In Situ Apoptosis Detection Kits, Trevigen, Gaithersburg, MD). Increased dark brown staining, evident of apoptosis, is seen in wild type spleens, lungs and kidneys. While the granzyme B null kidneys show apoptosis, there is no staining in the granzyme B null spleens and lungs. No apoptosis was noted in either set of heart and liver sections and is therefore not shown here. Photomicrographs were taken at 10X and 20X magnification. Apoptotic indexes, defined as the number of apoptotic cells per mm^2^, are shown for quantification of tissue apoptosis.

Spleen, lung, and kidney sections from wild type mice at 18 hours following CLP-induced polymicrobial sepsis (moderate model) were markedly TUNEL positive. (Figure 2) In contrast, spleens and lungs from septic granzyme B null mice lacked TUNEL staining. (Apoptotic index _WT vs. Granzyme B null_ for lung = 3,776±139 vs. 678±181 (p<0.001) and for spleen = 2,682±191 vs. 622±120 (p<0.001)). Kidneys stained positive for TUNEL in both wild type and granzyme B null animals while heart and liver did not stain in either strain. Adjacent sections stained for platelet antigen CD41 revealed similar abundant platelet accumulation in both lung and spleen of septic wild type and granzyme B null mice. (Figure 1D).

### Septic Platelets Induce Apoptosis in a Caspase-Mediated, Perforin-Independent Manner

**Figure 3:**
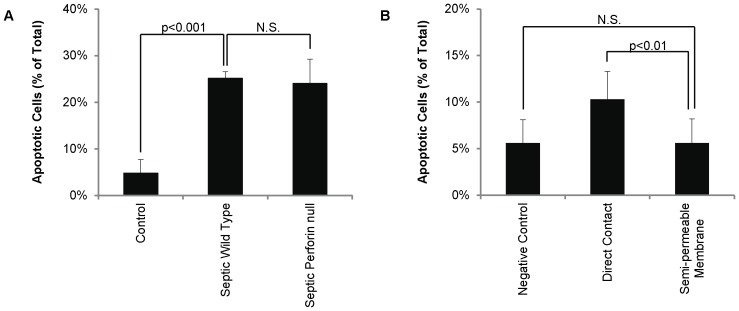
Platelet induced splenocyte apoptosis is perforin independent and contact dependent. A. Platelets harvested from septic mice induce apoptosis in control CD4^+^ splenocytes in the absence of perforin. Percent apoptosis was significantly higher in splenocytes co-incubated with platelets harvested from septic wild type (i.e. C57BL6) mice (n = 5) than with platelets from healthy wild type mice (n = 5) and splenocytes without platelets. Repeat experiments with platelets from septic perforin null mice showed no reduction in induced splenocyte apoptosis. **B.** Direct platelet contact is necessary for granzyme B-mediated apoptosis. Incubation across a dividing semi-permeable membrane reduced splenocyte apoptosis to a rate indistinguishable from non-platelet treated controls.

Granzyme B is known to target caspases in mice and humans [25] and Bid-induced mitochondrial cell death pathways in humans only [26]–[28]. To confirm septic platelets induce apoptosis via a mechanism consistent with granzyme B action in mice, we used platelet-splenocyte co-incubations as an *ex vivo* model for this interaction. At 18 hours post-CLP, platelets from septic wild type mice induced more splenocyte apoptosis *ex vivo* than platelets from healthy wild type mice (25.1±1.4% vs. 4.8±2.9%; p = 0.0004). (Figure 3A) The apoptotic splenocytes were almost entirely caspase positive (i.e. >98%).

When formed in cytotoxic lymphocytes and natural killer cells, granzyme B typically enters target cells through a channel of co-released perforin [25] but can also enter independently [29]. Therefore, we repeated the co-incubation experiments above with platelets from septic perforin null mice. In this condition, there was no change in percent-splenocyte apoptosis by septic perforin null platelets (24.0±5.2%) compared to septic wild type platelets. (Figure 3A).

### Platelets Require Direct Physical Contact with Splenocytes to Induce Apoptosis

To determine if platelets can induce end-organ apoptosis in the absence of direct contact with the target cells (implying a microparticle-mediated as opposed to directly-mediated process), septic platelets and healthy splenocytes were incubated as before, in suspension, or separated by a semi-permeable membrane. Incubation across a dividing semi-permeable (0.4 µm) membrane reduced splenocyte apoptosis (10.3±3.0 vs. 5.6±2.6; p<0.01) to a rate indistinguishable from non-platelet treated controls (5.6±2.5%; p = NS). (Figure 3B) As before, apoptotic splenocytes were almost entirely caspase positive (i.e. >98%).

### Platelet-Induced Splenocyte Apoptosis Is Blocked By GPIIb/IIIa Inhibition

**Figure 4:**
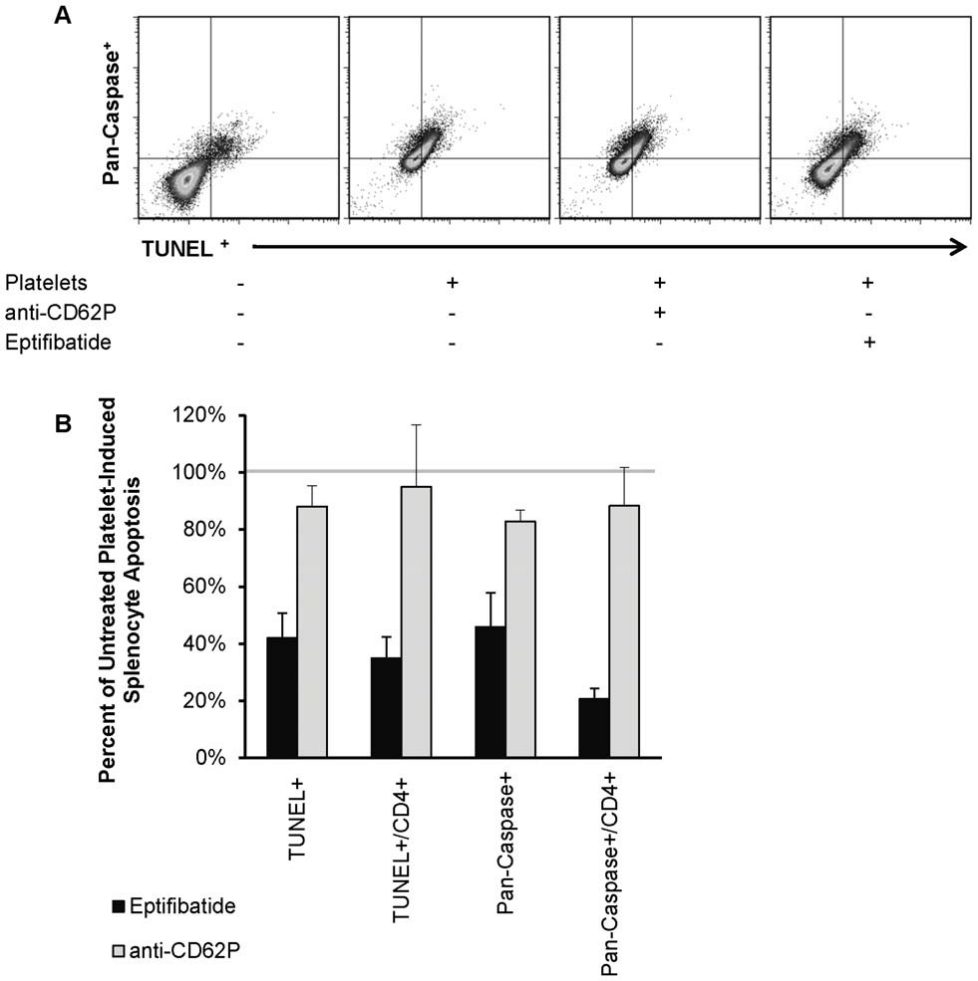
Eptifibatide reduces septic platelet-induced splenocyte apoptosis *ex vivo*. **A.** Representative flow cytometry staining of CD4^+^ splenocytes for pan-caspase FLICA (Y axis) vs. TUNEL (X axis) in presence of (left-to-right) no platelets, septic platelets, septic platelets with anti-62P antibody, or septic platelets with eptifibatide. **B.** Shown is the mean±SEM percent of septic platelet-induced splenocyte and CD4^+^ splenocyte apoptosis (i.e. TUNEL^+^ and pan-Caspase^+^) compared between pretreatment conditions (i.e. eptifibatide and anti-CD62P). These results were normalized to the level of apoptosis in splenocytes incubated with untreated septic platelets (solid line). Both splenocytes overall and CD4^+^ splenocytes showed a significant reduction (p<0.05) in apoptosis when platelets were pre-treated with eptifibatide. Pretreatment with an anti-CD62P monoclonal antibody did not significantly alter platelet-induced splenocyte apoptosis.

The finding that physical separation of septic platelets from splenocytes eliminated apoptosis raised the question whether pharmacologic separation would have the same effect. To that end, platelet aggregation was inhibited *ex vivo* with either a weak (anti-CD62P neutralizing antibody) or strong (eptifibatide) platelet aggregation inhibitor. Co-incubation of eptifibatide-exposed septic wild type platelets with healthy splenocytes significantly decreased splenocyte apoptosis overall and in the CD4^+^ fraction as compared to co-incubation with non-exposed septic platelets (overall = 66.5±10.6% reduction, p = 0.008; CD4^+^ = 85.0±20.7% reduction, p = 0.026). (Figure 4) No difference in apoptosis was observed for septic platelets pretreated with the anti-CD62P antibody.

### In vivo Eptifibatide Treatment Decreases Progression of Murine Sepsis

**Figure 5:**
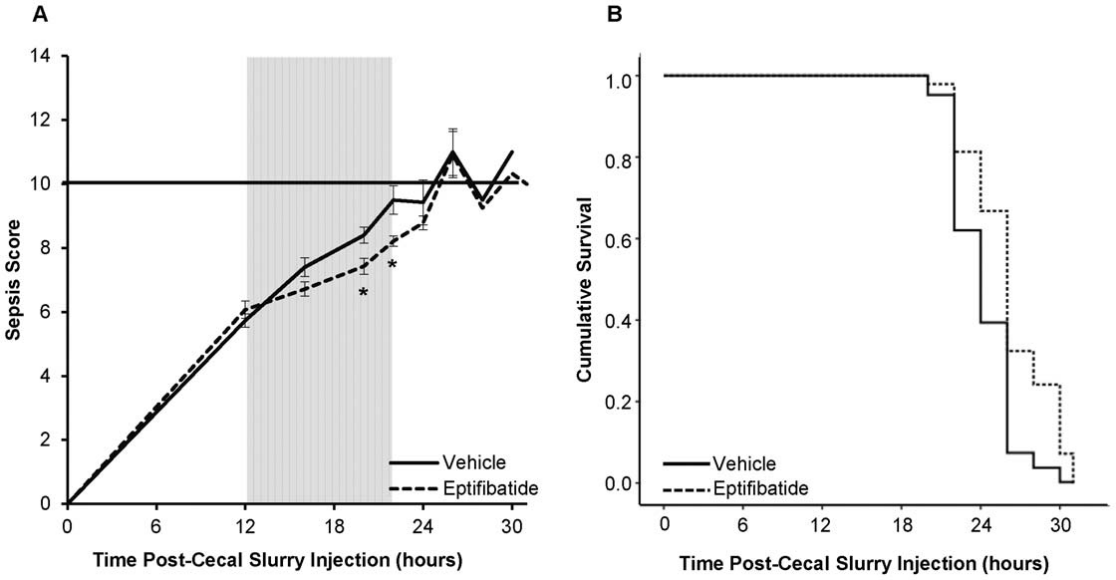
Sepsis survival and severity in vehicle and eptifibatide treated mice in a cecal slurry sepsis model. **A.** Eptifibatide treated mice had lower sepsis scores than vehicle treated mice at time points following drug administration. For example, at the 20 hour injection, the mean±SEM vehicle treated score was 8.4±0.3 while the eptifibatide treated score was 7.4±0.3 (p = 0.01) **B.** Kaplan-Meier survival curve for vehicle and eptifibatide treated mice in hours after cecal slurry injection. Eptifibatide treated mice survived longer following cecal slurry injection than vehicle treated mice (p = 0.019 by Cox Proportional Hazard Regression).

To assess the *in vivo* efficacy of GPIIb/IIIa blockade in sepsis, we treated septic mice (cecal slurry-induced) with an intravenous bolus of eptifibatide vs. PBS vehicle. Septic wild type mice treated with eptifibatide had lower sepsis scores during the period of anticipated treatment effect (i.e. 4 hours after each drug administration) than those treated with vehicle. (Figure 5A) In addition, Kaplan-Meier survival analysis showed that eptifibatide-treated mice survived longer than vehicle treated mice (p = 0.019 by Cox Proportional Hazard Regression) (Figure 5B).

## Discussion

Using experimental murine models of sepsis, we defined the site(s) of and mechanism(s) by which platelets induce end-organ apoptosis in sepsis. Platelet induced-apoptosis occurs in the spleen and at least one non-lymphoid organ, the lung. This granzyme B-mediated cytotoxicity requires direct contact between platelets and end-organ cells but is perforin independent. Further, we exploited the therapeutic potential of the contact-dependent nature of platelet-induced splenocyte apoptosis by markedly reducing *ex vivo* apoptosis with eptifibatide, a GPIIb/IIIa receptor inhibitor of platelet aggregation. *In vivo*, eptifibatide treatment improved clinical indicators and mortality in experimental sepsis. Collectively, these findings extend our previous work identifying platelet granzyme B-based cytotoxicity in septic humans and mice [10] and raise interesting questions regarding the role of GPIIb/IIIa blockade in sepsis.

Platelets are known to accumulate in both immune (spleen) [11] and non-immune (liver, lung, intestine) organs during sepsis [11]–[14]. Meanwhile, sepsis leads to apoptosis of both immune (lymphocytes) and non-immune (epithelial, endothelial, lung and intestine) cells [6]–[9], [30]. Lymphocyte apoptosis in sepsis is widespread, occurring in thymus, spleen, and gut-associated lymphoid tissues and has been shown to be associated with worse outcomes [31]–[33]. Increased levels of splenocyte apoptosis in particular reduce survival in animals after CLP [34], demonstrating the importance of our finding that absence of granzyme B leads to diminished splenocyte apoptosis. Herein we showed that sites of platelet aggregation (i.e. lung and spleen) also show increased levels of apoptosis in granzyme B containing, but not granzyme B null, mice. Prior to this, the coincident accumulation of platelets in failing organs in sepsis [12]–14,35 raised cause-and-effect questions. Our findings suggest platelets are causative in this relationship.

In addition to determining sites of platelet granzyme B-induced apoptosis in sepsis, we also determined vital mechanistic aspects of this process. In its typical role, in cytotoxic lymphocytes, granzyme B is stored and then released from secretory granules (also frequently containing perforin) upon synapse formation with virus infected or transformed target cells, leading to induction of apoptotic cell death pathways [36]. Whether platelet granzyme B-mediated apoptosis proceeds in a similar fashion was unknown. We showed that platelet granzyme B-mediated apoptosis is perforin independent and required direct contact between platelets and target cells. The requirement for direct contact between platelets and lymphocytes suggests that platelet-derived microparticles (which alone can be cytotoxic [15]–[17]) are not the primary initiator of this apoptosis.

The contact-dependent nature of platelet-induced splenocyte apoptosis led us to hypothesize that inhibitors of platelet aggregation could potentially decrease target cell apoptosis during sepsis. In fact, we demonstrated that septic platelet exposure to the anti-platelet compound, eptifibatide, reduces splenocyte apoptosis *ex vivo*. Eptifibatide functions as a reversible antagonist to the plasma membrane glycoprotein GPIIb/IIIa, which is found solely on platelets and platelet progenitor cells. GPIIb/IIIa belongs to a large class of cell surface receptors known as integrins, which take part in cell adhesion [37]–[40]. When platelets become activated, fibrinogen binds to multiple GPIIb/IIIa receptors, thereby bridging platelets and facilitating platelet aggregation. Eptifibatide, in particular, is an extremely effective inhibitor of platelet aggregation and is distinctive in the fact that it binds specifically to GPIIb/IIIa, with low affinity for other integrins [41].

With regard to sepsis, other GPIIb/IIIa antagonists and additional anti-platelet compounds have been studied in animal models and in certain cases have been shown to decrease coagulation activation and subsequent endothelial dysfunction and tissue injury during septic shock [42]–[45]. Our findings that pretreatment and subsequent co-incubation in the presence of eptifibatide decreases splenocyte apoptosis *in vitro* and *in vivo* treatment improves sepsis mortality have potentially important clinical implications. In fact, two human studies, both retrospective evaluations, have shown decreased mortality and decreased levels of MODS in examined adults admitted to the ICU who were already incidentally receiving anti-platelet compounds (either aspirin, clopidogrel or a combination of the two) [46], [47]. Additionally, clopidogrel, an ADP receptor antagonist, when given pre-hospitalization, is associated with a reduced incidence of acute lung injury in patients admitted to an intensive care unit [48]. Notable also was the fact that anti-platelet medications did not increase rates of bleeding. Collectively, these findings are in concordance with the decreased progression of sepsis we demonstrated in eptifibatide-treated mice with polymicrobial sepsis.

Anti-aggregation of platelets and splenocytes (i.e. reduced platelet-splenocyte contact) is only one possible mechanism by which eptifibatide may act in this scenario. Another possible mechanism is outside-in signaling, a mechanism by which extracellular binding to integrins activates intracellular signaling pathways [49], resulting in cytoskeletal rearrangements, and increased platelet granule release [50]. It is possible that eptifibatide inhibits the activation of intracellular signaling pathways, leading to a decrease in the release of granules, which may contain apoptosis promoting proteins such as granzyme B. This pathway’s ability to participate in sepsis and MODS warrants further investigation.

We acknowledge several limitations to our study. First, while we previously demonstrated that granzyme B is increased in human pediatric sepsis patients [10] and although CLP and cecal slurry are validated animal models, whether apoptosis progresses the same in mice as in humans is not clear. Second, in most cases our mice were sacrificed at 18 hours status post-CLP, thus we could be underappreciating changes that occur both before and after this time point. Additionally, our mice did not receive the intensive care interventions (antibiotics, mechanical ventilation, inotropes/vasopressors) that are standards of care in human patients. This reduces confounding variables in the model but limits our ability to generalize our data to a human sepsis population.

In summary, during sepsis, platelet granzyme B-mediated apoptosis occurs in spleen and lung tissue. This process proceeds in a perforin-independent, caspase-mediated, and contact-dependent manner, which can be inhibited by the GPIIb/IIIa inhibitor eptifibatide. In our preclinical experiments, the absence of granzyme B or treatment with eptifibatide resulted in less severe sepsis and extended survival. This builds on prior work demonstrating that granzyme B is upregulated in septic shock non-survivors [51] and further solidifies the important role played by this enzyme in platelets during sepsis. We have shown that inhibition of platelet aggregation via GPIIb/IIIa blockade slows progression of murine sepsis. The mechanism(s) by which this proceeds is unknown, but evaluating whether granzyme B-dependent end organ apoptosis contributes is a possibility that warrants further study.
